# Antitumor Activity of Chitosan-Coated Iron Oxide Nanocomposite Against Hepatocellular Carcinoma in Animal Models

**DOI:** 10.1007/s12011-022-03221-7

**Published:** 2022-07-22

**Authors:** Monda M. M. Badawy, Gehan R. Abdel-Hamid, Hebatallah E. Mohamed

**Affiliations:** 1 Department of Health Radiation Research, National Center for Radiation Research and Technology (NCRRT), Egyptian Atomic Energy Authority (EAEA), Cairo, Egypt; 2 Department of Radiation Biology, National Center for Radiation Research and Technology (NCRRT), Egyptian Atomic Energy Authority (EAEA), Cairo, Egypt

**Keywords:** Hepatocellular carcinoma, Iron oxide nanocomposite, Gamma radiation, PI3K/Akt/mTOR, MAPK

## Abstract

Hepatocellular carcinoma (HCC) is among the most prevalent and lethal cancers worldwide. Chitosan-coated iron oxide nanocomposite (Fe_3_O_4_/Cs) is a promising bio-nanomaterial for many biological applications. The objective of this research was to evaluate the anticancer efficacy of Fe_3_O_4_/Cs against HCC in animal models. Fe_3_O_4_ nanoparticles were prepared and added to chitosan solution; then, the mixture was exposed to gamma radiation at a dose of 20 kGy. Rats have received diethylnitrosamine (DEN) orally at a dose of 20 mg/kg body weight 5 times per week during a period of 10 weeks to induce HCC and then have received Fe_3_O_4_/Cs intraperitoneal injection at a dose of 50 mg/kg body weight 3 times per week during a period of 4 weeks. After the last dose of Fe_3_O_4_/Cs administration, animals were sacrificed. DEN induced upregulation of PI3K/Akt/mTOR and MAPK (ERK, JNK, P38) signaling pathways and inflammatory markers (TLR4, iNOS, and TNF-α). DEN also decreases cleaved caspase-3 and increases liver enzymes (ALT, AST, and GGT) activities. Administration of Fe_3_O_4_/Cs significantly ameliorated the above-mentioned parameters.

## Introduction

Hepatocellular carcinoma (HCC) is the most prevalent primary liver cancer and the fourth highest cause of cancer-related death worldwide [1]. HCC usually with poor prognosis. Over the previous few decades, the number of people diagnosed with HCC and dying from it has grown. Advanced HCC, on the other hand, has very few therapy choices [2]. Chemotherapy is the most common traditional cancer treatment, rather than local lesions that can be treated with surgery or radiotherapy. Anticancer medications, on the other hand, come with many negative side effects. Each medicine has its own set of side effects that might lead to patient noncompliance and a decline in quality of life. The production of reactive oxygen species (ROS) is one of the most common causes of unfavorable responses [3].

Recent advances in nanotechnology have provided a plethora of methods for fighting cancer with novel and effective therapeutic agents that avoid the drawbacks that standard medications have. Nanomaterials have been used as cancer therapeutics due to their ability to modulate autophagy [4]. Many research findings have shown that nanomaterials, particularly metallic nanoparticles, could be used to treat cancer by promoting a variety of events such as mitochondrial damage, lysosome impairment, endoplasmic reticulum stress, and signalling pathway alterations, which all result in mitophagy stimulation, oxidative stress, and autophagic cell death. Importantly, as compared to noncancerous cells, metallic nanoparticles have shown intrinsic selectivity in triggering autophagy in cancer cells. Metal-based nanomaterials, on the other hand, may play conflicting roles in cell fate, inducing pro-survival autophagy in both cancer and normal cells [5–7]. As a result, inhibiting autophagy could be a realistic strategy for improving cancer therapy efficacy. Magnetic iron oxide nanoparticles (Fe_3_O_4_ NPs) are a type of multifunctional nanomaterial that is increasingly being used in a variety of biomedical fields, such as magnetic resonance imaging, magnetic hyperthermia, magnetic targeting, magnetic separation, biological catalysis, photo-responsive therapy, and drug delivery. They are currently widely used in tumor diagnosis and treatment [8]. Because of its low toxicity in biological systems, iron oxide nanoparticles have shown considerable promise in biomedical applications. Iron oxide nanoparticles’ magnetic and semiconductor characteristics may also lead to multifunctional medical uses [9]. Other advantages of Fe_3_O_4_ NPs include prolonged blood circulation, rapid clearance, low side effects, good imaging, and therapeutic efficacy [10]. The key advantage of these nanoparticles over conventional drug delivery systems is their excellent biodistribution in the body [9]. Furthermore, Fe_3_O_4_ NPs have catalytic activity similar to peroxidase and have been proposed as a mimic enzyme for cancer therapy via the well-known Fenton reactions, which can catalyse endogenous hydrogen peroxide (H_2_O_2_) into the hydroxyl radical (•OH) with high cytotoxicity and cause tumor cell death [11].

Chitosan is reported to have a high biodegradability, biocompatibility, and stability, as well as minimal toxicity and immunogenicity in cells [12]. Chitosan is also a mucoadhesive cationic polymer that has been used widely in recent years to deliver anticancer chemotherapeutics to tumor cells [13]. Chitosan’s amine group will promote solubility and hemocompatibility in the intracellular environment [12]. Furthermore, the positive surface of nanoparticles will interact with many cellular components, which is a characteristic of chitosan, resulting in a longer connection between the encapsulated component and the cells. The presence of chitosan on the particle surface may also aid in the opening of tight intracellular junctions, thereby increasing cellular absorption and hence therapeutic efficiency [14, 15]. Fe_3_O_4_/Cs has potentially better biomedical properties than uncoated Fe_3_O_4_ NPs; thus, it is a promising bio-nanomaterial for many biological applications [16].

The actions of the phosphatidylinositol 3 kinase/kinase Akt/mammalian target of rapamycin (PI3K/Akt/mTOR) pathways trigger autophagy. mTOR is a master regulator of cellular metabolism that responds to a variety of extracellular cues such as nutrition, growth hormones, and stress [17]. Its signalling pathway starts with insulin-activating PI3K, then moves on to Akt, which then activates mTOR [18]. Changes in cellular pathway proteins like PI3K/Akt/mTOR and MAPK/ERK, which are extensively dysregulated in malignant tumors, also support malignant cells’ ability to avoid apoptotic death and contribute to chemotherapy resistance [19]. Various types of NPs have been found to influence components in the mTOR signalling pathway and other related pathways, such as the AMPK and ERK pathways [20].

Towards this purpose, Fe_3_O_4_/Cs was successfully prepared and evaluated as anticancer against HCC in animal models.

## Material and Methods

### Materials

Diethylnitrosamine (DEN) purchased from Sigma-Aldrich (cat# N0756, St. Louis, MO, USA). Chitosan, ferrous chloride tetrahydrate (FeCl_2_. 4H_2_O), ferric chloride hexahydrate (FeCl_3_. 6H_2_O), and A sodium hydroxide (NaOH) were purchased from Merck. All chemicals were guaranteed or analytic grade reagents commercially available and used without further purification.

### Preparation of Fe_3_O_4_ Nanoparticles Coated with Chitosan

Fe_3_O_4_ nanoparticles were prepared by co-precipitating the Fe3 + and Fe2 + ions by, firstly, dissolving 4 gm of (FeCl_3_. 6H_2_O) and 2 gm of (FeCl_2_. 4H_2_O) in 200 ml of doubly distilled water by vigorous stirring on magnetic stirrer; then 200 ml of sodium hydroxide (0.5 M) was added drop wisely and very slowly under stirring and heating to 80 °C until complete precipitation obtained [21]. Heating was continued at 80°for another 30 min, and then dispersion of formed magnetic nanoparticles washed several times with water by decantation to remove any excess sodium hydroxide. One hundred milliliter of the prepared Fe_3_O_4_ nanodispersion was added to 100 ml of chitosan solution at a concentration of 1% wt/v and vigorously stirred under heating at 80 °C for 1 h; then the mixture exposed to gamma radiation at a dose of 20 kGy. The formed magnetic nanoparticles that coated with chitosan exposed to gamma radiation to initiate partial degradation of chitosan chains and to facilitate the coating process of Fe_3_O_4_ NPs by chitosan.

### Characterizations of Fe_3_O_4_/Chitosan Nanodispersions

#### Transmission Electron Microscopy (TEM)

TEM was used to observe the morphology (size and shape) of the formed magnetic nanoparticles. A drop of the resultant dispersion of magnetic nanoparticles mixture was deposited on an ultrathin carbon supported Cu grid and air-dried. Energy-filtered electron powder diffraction used TEM JEOL: JEM-100cx.

#### The X-Ray Diffraction (XRD)

The XRD analysis was performed using XD-DI Series, Shimadzu apparatus using nickel-filtered and Cu-K target, available in NCRRT.

### Determination of the Median Lethal Dose (LD50) of Fe_3_O_4_/Cs

Fe_3_O_4_/Cs were given intraperitoneally to male albino rats in doses ranging from 50 to 700 mg/kg. Mortality after 24 h was registered.

The LD50 of Fe_3_O_4_/Cs was calculated according to the formula [22].

$$\mathbf{LD}\mathbf{50}\boldsymbol=\mathbf{Dm}\boldsymbol-\boldsymbol\Sigma\boldsymbol(\mathbf{Zxd}\boldsymbol)\boldsymbol/\mathbf n$$

where *Dm* is the minimum dose which kills all animals in the group; *Z* is the mean of dead animals in two successive groups; *d* is the constant factor between two successive groups; *n* is the number of animals of each group; and *Σ* is the sum of (Zxd).

### Experimental Design

#### Animal Groups

Twenty-four male albino rats (weighing 120–150 g at the start of the experiment) were randomly divided into 4 groups (6 animals/group):

- **Group 1 (Control)**: Healthy animals do not receive any treatment.
- **Group 2 (DEN):** Rats were received diethylnitrosamine (DEN) orally at a dose of 20 mg/kg body weight, five times a week during a period of 10 weeks [23].
- **Group 3 (Fe**_**3**_**O**_**4**_**/Cs):** Rats were received 50 mg/kg body weight of chitosan-coated iron oxide nanocomposite (Fe_3_O_4_/Cs) suspended in distilled water by intraperitoneal injection three times a week during a period of 4 weeks [24].
- **Group 4 (DEN + Fe**_**3**_**O**_**4**_**/Cs):** Rats were received DEN orally as group 2 then treated with Fe_3_O_4_/Cs as group 3.

Rats were obtained from the National Centre for Radiation Research and Technology (NCCRT), Cairo, Egypt. The rats were housed in cages and maintained a 12-h light/dark cycle. They were allowed to acclimatize to the environmental conditions for 1 week before starting the experiment and were kept on standard food pellets containing all nutritive elements and liberal water ad libitum. All the ethical protocols for animal treatment were approved by the Ethical Committee at the National Center for Radiation Research and Technology (no: 3 A/ 22).

After the last dose of Fe_3_O_4_/Cs administration, rats were fasted overnight. Blood samples were withdrawn from the heart of each animal, under light anesthesia by diethyl ether. Blood was allowed to coagulate and then was centrifuged at 3000 rpm for 15 min, and the serum was separated in another tube. Immediately after blood sampling, animals were sacrificed by cervical dislocation; liver tissues were rapidly removed, washed in ice-cold saline, plotted to dry, and then were kept at − 80 °C till the day of analysis. Another part of liver tissue was placed in 10% formalin prepared in phosphate buffered saline (PBS) to be used for histopathological examination.

#### Molecular Investigation

##### Western Immunoblotting Analysis of PI3K/AKT/ mTOR and MAPK (P38, ERK1/2 and JNK) Proteins in Liver Tissue Homogenate

Liver tissue protein was extracted using TRIzol reagent, and protein concentration was quantified according to Bradford [25]. Twenty microgram of protein per lane was separated by sodium dodecyl sulfate–polyacrylamide gel electrophoresis (SDS-PAGE) using 10% acrylamide gels and transferred on to PVDA membranes. Membranes were incubated at room temperature for 2 h with blocking solution (5% non-fat dried milk in 10 mMTris-HCl, pH 7.5, 100 mMNaCl, and 0.1% Tween 20) and then incubated overnight at 4 °C with primary antibody towards targeted proteins with β-actin as a control. After washing three times in washing buffer (10 mMTris-HCL, pH 7.5, 100 mMNaCl, and 0.1% Tween 20), membrane was incubated with the secondary monoclonal antibody conjugated to horseradish peroxidase at room temperature for 2 h, and then membranes were washed four times with the same washing buffer. Membrane was developed and visualized by chemiluminescence using Invitrogen™ detection kit (Catalog #AHO1202) according to the manufacturer’s protocols and then exposed to X-ray film. Quantification of PI3K/AKT/ mTOR and MAPK (P38, ERK1/2 and JNK) proteins was carried out using scanning laser densitometer (Biomed Instrument Inc., USA).

##### Determination of Toll-Like Receptor 4 (TLR4) and Inducible Nitric Oxide Synthase (iNOS) Gene Expression

Total RNA was extracted from liver tissue using RNeasy Mini Kit (Qiagen, Cat. No. 74104) according to the manufacturer’s instructions. First strand complementary DNA (cDNA) synthesis was performed using QuantiTect Reverse Transcription Kit (Qiagen, Cat. No. 205311) according to the manufacturer’s instructions using 1 μg RNA as a template. RT-PCR were performed in a thermal cycler step one plus (Applied Biosystems, USA) using the Sequence Detection Software (PE Biosystems, CA). The oligonucleotides utilized in these experiments are listed in Table 1. The reaction mixture of total volume 25 μl was consisting of 2X SYBR Green PCR Master Mix (Qiagen, Cat. No. 204143), 900 nM of each primer and 2 μL of cDNA. PCR thermal-cycling conditions included an initial step at 95 °C for 5 min; 40 cycles at 95 °C for 20 s, annealing at 52 °C or 57 °C or 60 °C (as in Table 1) for 30 s, and 72 °C for 30 s. The relative expression of the real-time reverse transcriptase PCR products was determined by the ΔΔCt method. This method calculates a relative expression to housekeeping gene using the equation: fold induction = 2^−(ΔΔCt)^, where ΔΔ Ct = Ct [gene of interest (unknown sample)—Ct housekeeping gene (unknown sample)]—[Ct gene of interest (calibrator sample)—Ct housekeeping gene (calibrator sample)] [26].

**Table 1:** Primer sequences for the genes amplified

Gene	Strand	Sequence 5′—3′	Product length (bp)	Annealing temperature (°C)	Ref. Seq
TLR4	F	ATGAGGACTGGGTGAGAAAC	161	52	NM_019178
	R	CACCACCACAATAACTTTCC			
iNOS	F	CTCACTGTGGCTGTGGTCACCTA	101	60	NM_012611
	R	GGGTCTTCGGGCTTCAGGTTA			
β-actin	F	GATCAAGATCATTGCTCCTCCTGA	170	57	NM_031144
	R	CAGCTCAGTAACAGTCCGCCT			

#### Biochemical Investigation

Tumor necrosis factor alpha (TNF-α) and cleaved caspase-3 were measured in liver tissue homogenates, according to the manufacturer’s instructions using ELISA kits supplied by MyBioSource, Inc. P.O. Box 153,308 San Diego, CA 92,195–3308, USA, while alanine aminotransferase (ALT), aspartate aminotransferase (AST), and gamma-glutamyl transferase (GGT) activities were analyzed in serum using commercial kits provided by Spinreact (Spain).

#### Histopathological Examination

Liver tissue specimens were fixed in 10% formol saline, then trimmed off, and washed and dehydrated in ascending grades of alcohol. The dehydrated specimens were then cleared in xylene, embedded in paraffin blocks, and sectioned at 4–6 µm thick. The obtained tissue sections were deparaffinized using xylol and stained using hematoxylin and eosin (H&E) for histopathological examination through the electric light microscope [27].

##### Histological Grade of Hepatocellular Carcinoma

The most widely used grading system in HCC (Edmondson and Steiner system). Grade I consists of small tumor cells, arranged in trabeculae, with abundant cytoplasm and minimal nuclear irregularity that are almost indistinguishable from normal liver tissue. Grade II tumors have prominent nucleoli, hyperchromatism, and some degree of nuclear irregularity. Grade III tumors show more pleomorphism than grade II and have angulated nuclei. Grade IV tumors have prominent pleomorphism and often anaplastic giant cells.

**The pathological response of HCC to therapy was graded as Galal et al. (2013)** [28]**.**

**Table:** 

Grade	Pathological response
Complete	100% tumor necrosis
Partial	51–99% tumor necrosis
Poor	< 50% tumor necrosis

### Statistical Analysis

The data were presented as means ± standard error of mean (S.E.); they were analyzed using one-way ANOVA followed by Tukey–Kramer multiple comparison test. The Graph Prism software, version 5, Inc., USA, was used to perform the statistical analysis and graphical presentations. The level of significance was fixed at *P* ≤ 0.05 with respect to all statistical tests.

## Results

### *Characterization of Fe*_*3*_*O*_*4*_*/Cs*

#### Transmission Electron Microscopy (TEM)

**Fig. 1:**
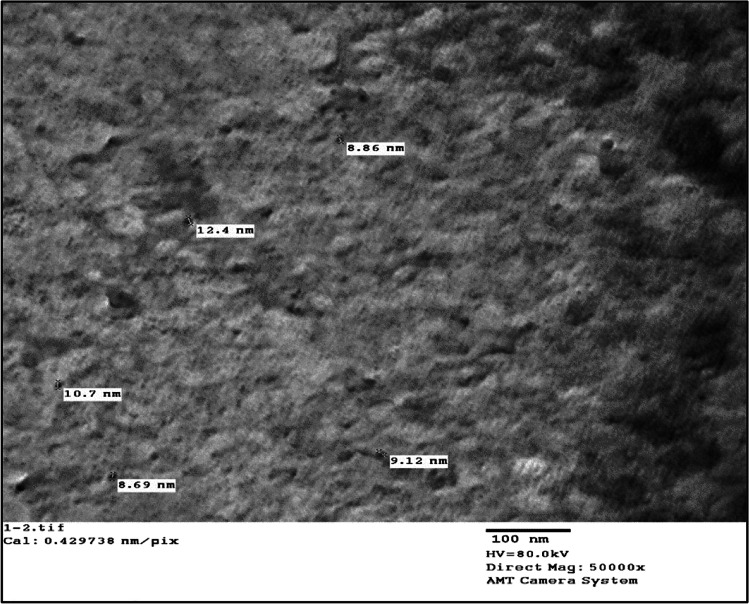
TEM micrograph for the Fe_3_O_4_/Cs

Figure 1 depicts a typical TEM micrograph of Fe_3_O_4_/chitosan nanoparticles. Because chitosan inhibits nanoparticle aggregation and consequently helped in uniform dispersion of the nanoparticles, Fe_3_O_4_/chitosan nanoparticles showed virtually little dispersion, with a mean diameter of about 9.95 nm. This might be due to the reaction taking place exclusively on the surface of the particle, and hence the attempt to make monodispersed Fe_3_O_4_/chitosan nanoparticles in this study was successful.

#### The X-Ray Diffraction (XRD)

**Fig. 2:**
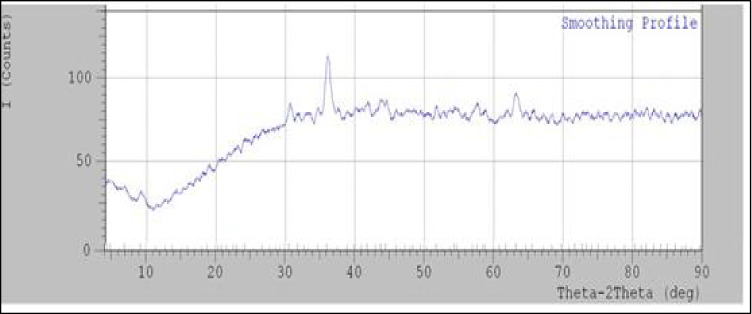
the XRD of Fe_3_O_4_/Cs

Figure 2 shows the XRD of Fe_3_O_4_/Cs, which confirms the formation of Fe_3_O_4_/Chitosan nanoparticles through appearance of the characteristic peaks related to Fe_3_O_4_ nanoparticles at 2-Theta: 35 °C, 42 °C, 58, and 63. The appearance of the broad peak in the 2-Theta range 20–30° is related to the polymeric part of chitosan. Also, the coating of magnetic nanoparticles with chitosan chains causes a noticeable change in the XRD base line of Fe_3_O_4_ NPs (Fig. 2).

### The Median Lethal Dose (LD50) of Fe_3_O_4_/Cs

The results revealed that the LD50 was found to be 500 mg/kg body weight for the Fe_3_O_4_/Cs for the intraperitoneal administration (Table 2). One-tenth of the LD50 value has been used as an ideal dose to determine the in vivo antitumor activity of Fe_3_O_4_/Cs.

**Table 2:** Calculation of the median lethal dose (LD50) of Fe3O4/Cs

Dose of Fe_3_O_4_/Cs (mg/kg body weight)	Number of animals	Number of dead animals	*Z*	*d*	(*Z*) × (*d*)
50	6	0	0	50	0
100	6	0	0	50	0
200	6	0	0	100	0
300	6	0	0	100	0
400	6	2	1	100	100
500	6	3	2.5	100	250
600	6	4	3.5	100	350
700	6	6	5	100	500

LD50 = 700 − (1200/6) = 700 − 200 = 500 mg/kg

### Molecular and Biochemical Studies

#### ***The Effect of Fe***_***3***_***O***_***4***_***/Cs on PI3K/AKT/ mTOR and MAPK (P38, ERK1/2 and JNK) Pathways***

Treatment with DEN manifested a significant (*P* ≤ 0.05) increase in the expression of the PI3K, AKT, and mTOR signal pathways (PI3K, 4.2-fold; AKT, 1.9-fold; and mTOR, 3.7-fold in respect to normal control). Treatment with Fe_3_O_4_/Cs induces a remarkable reduction in the expression of (PI3K, − 0.54-fold; AKT, − 0.57-fold; and mTOR, − 0.53-fold in respect to DEN) as shown in Table 3.

**Table 3:** The effect of Fe_3_O_4_/Cs on PI3K/AKT/ mTOR and MAPK (P38, ERK1/2, and JNK) pathways

	PI3K	AKT	mTOR	P38	ERK1/2	JNK
Control	1.02 ± 0.01	1.02 ± 0.01	1.00 ± 0.04	1.01 ± 0.01	1.04 ± 0.02	1.04 ± 0.04
Fe_3_O_4_/Cs	1.03 ± 0.02	1.06 ± 0.03	1.00 ± 0.01	1.04 ± 0.03	1.01 ± 0.01	1.03 ± 0.01
DEN	5.30 ± 0.85^a^	2.95 ± 0.21^a^	4.70 ± 0.21^a^	5.13 ± 0.01^a^	5.07 ± 0.05^a^	3.65 ± 0.21^a^
DEN + Fe_3_O_4_/Cs	2.42 ± 0.54^b^	1.27 ± 0.33 ^b^	2.20 ± 0.14^a,b^	2.10 ± 0.42^a,b^	3.55 ± 0.35^a,b^	1.30 ± 0.14^b^

Each value represents the mean ± standard deviation.

^a^Significant difference versus control group at *P* ≤ 0.05.

^b^Significant difference versus DEN group at *P* ≤ 0.05.

**Fig. 3:**
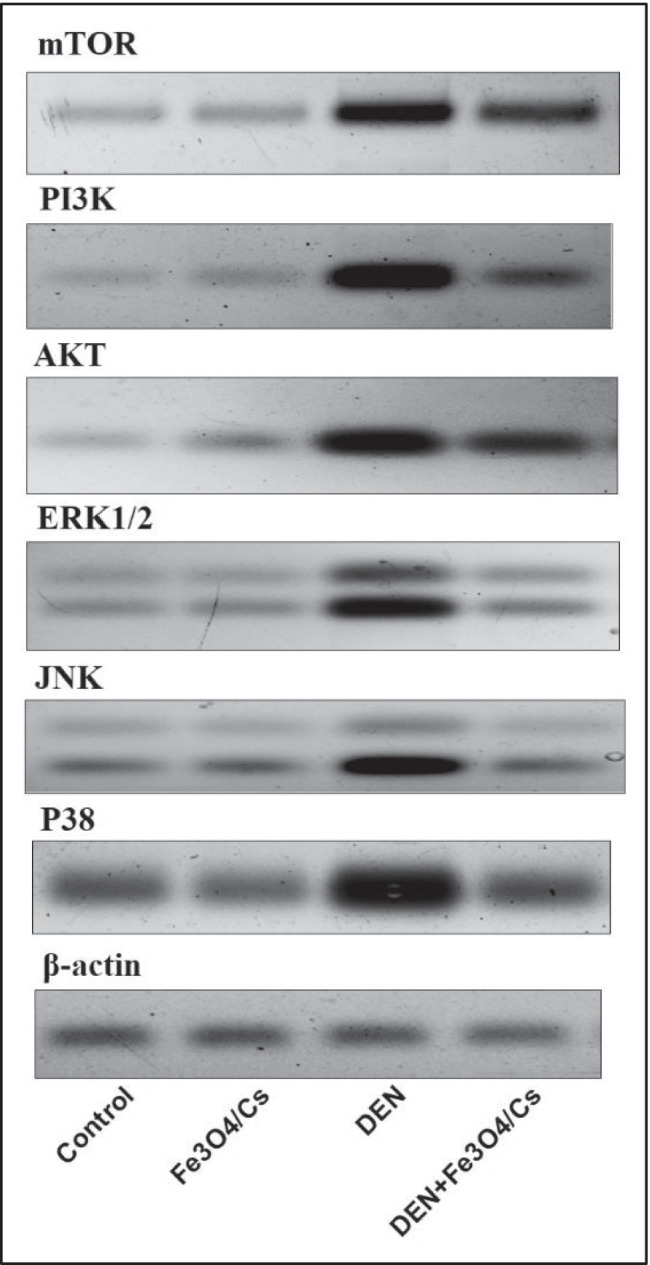
The effect of Fe_3_O_4_/Cs on PI3K/AKT/ mTOR and MAPK (P38, ERK1/2 and JNK) protein expression

Moreover, protein expression of the mitogen-activated protein kinase (MAPK) family members shows a significant (*P* ≤ 0.05) increase (p38 MAPK, 4.08-fold; ERK1/2, 3.88-fold; JNK, 2.51-fold in DEN-treated rats in respect to normal control). Treatment with Fe_3_O_4_/Cs for DEN + Fe_3_O_4_/Cs group induces a remarkable reduction in these proteins (p38 MAPK, − 0.59-fold; ERK1/2, − 0.29-fold; JNK, − 0.64-fold in respect to DEN group) as shown in Table 3 and Fig. 3.

#### The Effect of Fe_3_O_4_/Cs on TLR4, iNOS, TNF-α, and Cleaved Caspase-3

**Fig. 4:**
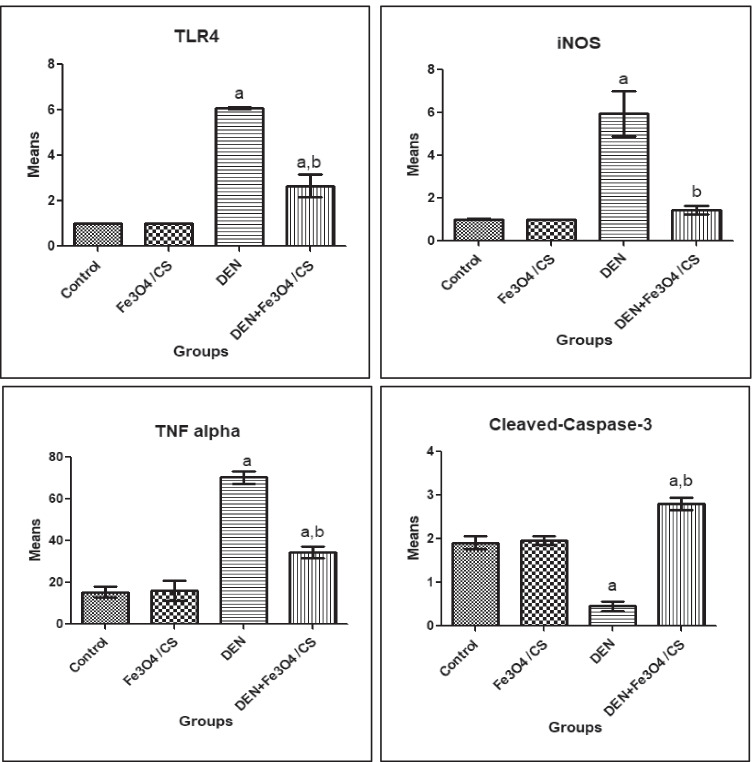
The effect of Fe_3_O_4_/Cs on the inflammatory biomarkers (TLR4 and iNOS), TNF-α, and cleaved caspase-3. ^a^Significant difference versus control group at *P* ≤ 0.05; ^b^significant difference versus DEN group at *P* ≤ 0.05

The evaluation for the anti-inflammatory activity of Fe_3_O_4_/Cs was shown in Fig. 4; the data revealed a high significant (*P* ≤ 0.05) increase in the levels of the inflammatory biomarkers, TLR4 and iNOS (5.01-fold and 4.83-fold), respectively, in DEN-treated rats as compared to the corresponding control. In addition, the data displayed a high significant (*P* ≤ 0.05) increase in the levels of TNF-α (3.57-fold) with significant decrease in cleaved caspase-3 activity (− 0.76-fold) in DEN-treated rats as compared to the corresponding control. Meanwhile, the data displayed a dramatic decrease in (TLR4, − 0.56-fold; iNOS, − 0.76-fold; TNF-α, − 0.51-fold) with significant increase in the cleaved caspase-3 activity (5.1-fold) for Fe_3_O_4_/Cs + DEN treatment group as compare to DEN group.

#### The Effect of Fe_3_O_4_/Cs on Liver Function Biomarkers

**Fig. 5:**
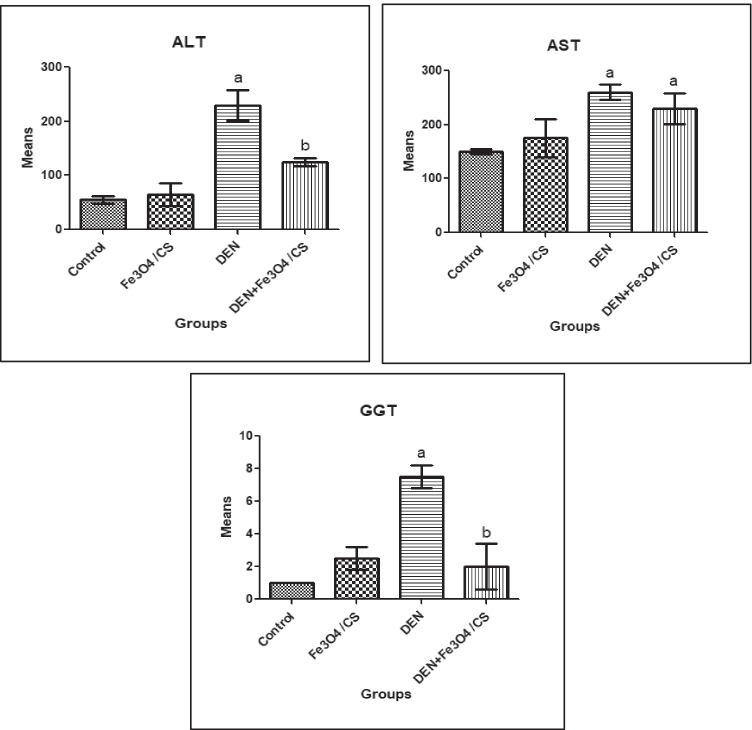
The effect of Fe_3_O_4_/Cs on liver function biomarkers. ^a^Significant difference versus control group at *P* ≤ 0.05; ^b^significant difference versus DEN group at *P* ≤ 0.05

The levels of ALT, AST, and GGT in the serum of all groups were examined to see if Fe_3_O_4_/Cs had any influence on liver function biomarkers. As shown in Fig. 5, DEN treatment caused severe liver injury, as evidenced by a significant (*P* ≤ 0.05) increase in serum (ALT, 3.2-fold; AST, 0.73-fold; and GGT, 6.5-fold for DEN group vs. control group), which was mitigated by Fe_3_O_4_/Cs treatment (ALT, − 0.46-fold; AST, − 0.12-fold; and GGT, − 0.73-fold for Fe_3_O_4_/Cs + DEN group vs. DEN group.

## Histopathological Observation.

**Fig. 6:**
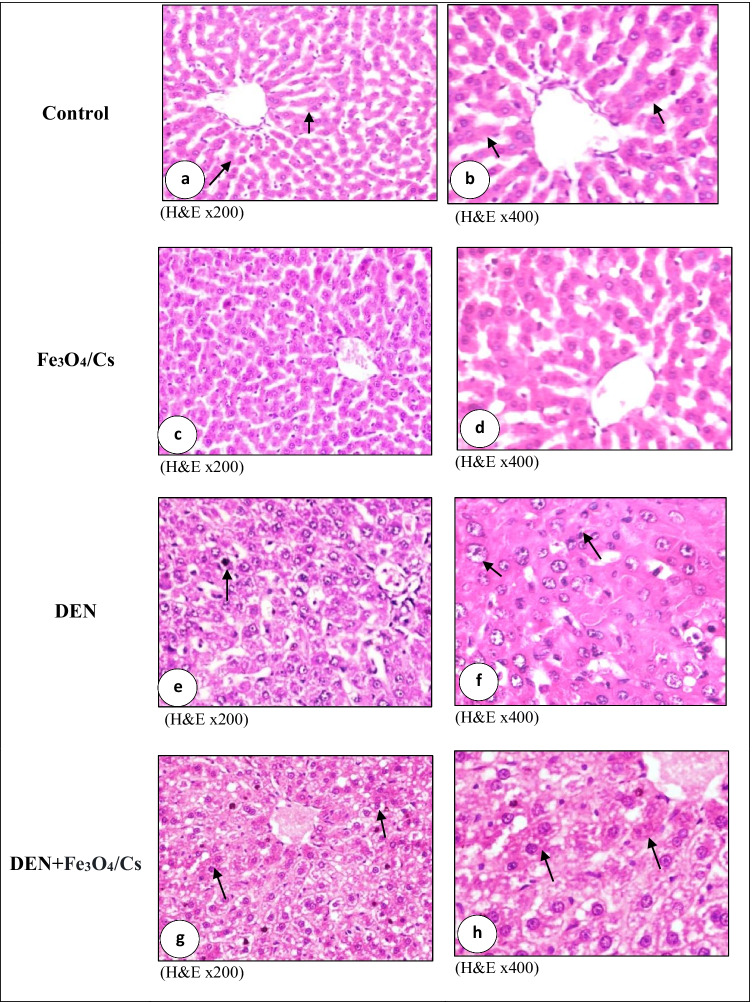
Photomicrograph of liver showing **a** normal histological structure of hepatic lobules arrow, **b** normal large polygonal cells with prominent round nuclei arrow, **c** normal histological structure of hepatic lobules, **d** normal large polygonal cells with prominent round nuclei, **e** deeply basophilic malignant cells with few mitotic figure arrow, **f** nuclear pleomorphism arrow and leukocytic infiltration, **g** necrobiotic changes of hepatic carcinoma cells with hyperplasia of Kupffer cells arrow, and **h** partial apoptosis and deeply eosinophilic cytoplasm without obvious mitotic figures arrow

Light microscopic observation for control group demonstrated control hepatic tissue included normal polygonal cells with noticeable round nuclei and eosinophilic cytoplasm, as well as a few spaced hepatic sinusoids distributed in between the hepatic cords with fine Kupffer cell arrangement (Fig. 6a−b). Animals group treated with (Fe_3_O_4_/Cs) only showed normal histological picture similar to normal control group without any pathological alterations (Fig. 6c−d).

Histopathological alterations of liver tissue sections of hepatocellular carcinoma-induced rat group by (DEN) revealed polyhedral to round hepatocytes with dense, centrally located vesicular nuclei. The neoplastic areas showed poorly differentiated large cells with hyperchromatic nuclei and prominent nucleoli. The neoplastic cells showed pleomorphism, deeply basophilic scanty cytoplasm, and few mitotic figures (grade II). The hepatic lobule displayed disorganization of hepatic cords with hyperplasia of Kupffer cells (Fig. 6e−f).

Carcinogenic animals group treated by (Fe_3_O_4_/Cs) revealed necrobiotic changes of hepatic carcinoma cells with hyperplasia of Kupffer cells. Partial 51–99% tumor necrosis was noticed. Apoptosis and deeply eosinophilic cytoplasm without obvious mitotic figures were seen. Disorganization of carcinoma cells with widening of hepatic sinusoids were notice. Leukocytic infiltration mainly lymphocytes and macrophages were seen. Numerous numbers of binucleated cells and karyomegaly with peripheral condensation of its chromatin were noticed (Fig. 6g−h).

## Discussion

HCC is a worldwide issue. HCCis the sixth common cancer worldwide, while it is fourth common cancer in Egypt. Egypt is Africa’s third and the world’s 15th most populous country, for HCC [29]. Iron oxide nanoparticles possess hopes in nanomedicine because of their potential use in cancer therapy, drug transport, and bioimaging [30]. Because Fe_3_O_4_ nanoparticles are easy to combine and oxidize, they are frequently coated to achieve better characteristics for targeted drug/gene delivery [7]. In view of these considerations the aim of the current study was to prepare and evaluate the efficacy of Fe_3_O_4_/Cs as an anticancer agent.

Fe_3_O_4_/Cs were prepared by exposure to 20 kGy gamma radiation then characterized using TEM and XRD. Gamma radiation is favored method for metallic nanoparticles synthesis because it is reproducible, simple, inexpensive, and uses fewer toxic precursors, as well as may control the yield shape of the monodispersed metallic nanoparticles [31]. Prepared Fe_3_O_4_/Cs showed a typical TEM micrograph for monodispersed Fe_3_O_4_/chitosan nanoparticles as chitosan inhibited the aggregation of nanoparticles. XRD showed characteristic peaks related to Fe_3_O_4_ nanoparticles (at 2-Theta: 35 °C, 42 °C, 58, and 63) and broad peak (in the 2-Theta range 20–30°) in related to the polymeric part of chitosan. The peak positions were unchanged, which illustrated that the chitosan binding process did not result in the phase change of Fe_3_O_4_ [32]. Prepared Fe_3_O_4_/Cs were evaluated as anticancer agent for HCC DEN-induced rats, where PI3K/Akt/mTOR and MAPK (ERK, JNK, P38) signaling pathways were studied, as well as inflammatory markers (TLR4, iNOS and TNF-α), cleaved caspase-3, and liver enzyme (ALT, AST and GGT) activities.

The PI3K/AKT/mammalian target of rapamycin (mTOR) signalling pathway is one of the most essential intracellular pathways, and it can be regarded a key regulator for cancer [33]. PI3K/AKT/mTOR pathway activation leads to tumor growth and anticancer drug resistance [34]. Growth factors activate the PI3K/Akt/mTOR pathway by connecting to their receptors and activating the substrates of the receptors. The phosphoinositide 3-kinase (PI3K) then binds to the active receptor’s intracellular part and transforms phosphatidylinositiol-4, 5-phosphate (PIP2) to phosphatidylinositiol-3, 4, 5-phosphate (PIP3). PIP3 then stimulates the PDK1 to AKT pathway, causing mTOR to be activated [35]. PI3K/Akt/mTOR and MAPK are more frequently activated intracellular pathways and the best characterized in HCC. Possibly playing a role in its pathogenesis [36]. In the current study, the DEN-challenged group had higher levels of PI3K/Akt/mTOR and MAPK pathway expression. Previous studies had found similar findings [37, 38]. Nanoparticle-based mTOR targeted therapy appears to be a promising treatment option for a variety of malignancies [39]. It’s worth mentioning that a large number of studies suggests that different NPs control mTOR activation, causing cell cycle arrest in cancerous cells [40], which simultaneously has an effect on the related signaling pathways AMPK and ERK. It is also worth noting that NP-mediated autophagy is correlated to the production of reactive oxygen species (ROS), which suppresses the mTOR pathway [41]. The Fe_3_O_4_ nanoparticles inhibited the mTOR phosphorylation [42] and AKT [43]. In the present study, Fe_3_O_4_/Cs modulated diethylnitrosamine (DEN)-induced HCCin animals models via PI3K/Akt/mTOR and MAPK (ERK, JNK, and P38) Signaling pathways. Such results were reported by the previous studies [41, 42, 44].

Toll-like receptor (TLR-4) appears to have a role in hepatocarcinogenesis, as TLR-4 expression increased as chronic hepatitis progressed to cirrhosis and then to HCC. TLR-4 expression was associated to a larger tumor size and a higher HCC grade, suggesting that it could be used to predict HCC prognosis. TLR-4 expression was found to be significantly higher in the DEN-treated group in the current investigation. Prior studies reported similar findings [45]. The effectiveness of metal oxide nanoparticles for TLRs varies depending on the metal type. Iron oxide nanoparticles (IONPs) differentially modulate TLR ligand-induced cytokine levels. The interactions between IONPs and TLR ligands are complicated and vary depending on the TLR ligand, nanoparticle size, and interactions between nanoparticles and TLR ligands [46]. Downstream TLR signaling causes MAPK cascades activation [47]. When these pathways are activated, macrophages undergo metabolic and functional changes, including differential expression of pro-inflammatory cytokines like tumor necrosis factor (TNF-α) [48]. In the current study, we observed upregulation in iNOS expression and increasing in TNF-α level in rats treated with DEN. Such results agree with the previous study done by Fathy and Nikaido, (2013) [49]. Metal oxide nanoparticles have been reported to induce both immunosuppressive and anti-inflammatory responses [50, 51]. According to the current findings, Fe_3_O_4_/Cs modulated the TLR4 activation. Also, Fe_3_O_4_/Cs treatment causes significant decreases in TNF-α level and downregulation in the expression of iNOS. Such results were reported by previous studies [46, 50, 52, 53].

Caspase-3 is a key molecule in cancer cell death. The active form of caspase-3, cleaved caspase-3, was the major cleavage enzyme that promoted apoptosis [54]. In the present study, we observed inhibition in cleaved caspase-3 activity in rats treated with DEN, which agree with the results reported by Lin et al. (2017) [55]. Fe_3_O_4_ nanoparticles induce apoptosis in cancer cells by raising the quantity of reactive oxygen species (ROS) and intracellular calcium, as well as boosting the expression of caspase-3 and caspase-9 and decreasing the expression of Bcl-2, as well as causing direct DNA damage [56]. The expression of cleaved caspase-3 is increased by the Fe_3_O_4_/Cs in the current study. Previous investigations had reported similar findings [57, 58].

Cytoplasmic transaminases, including ALT and AST, are released and circulated in response to DEN stimulation. Both ALT and AST are useful diagnostic markers for liver damage [59]. The data showed that the activities of ALT, AST, and GGT increased significantly, indicating that the DEN-induced HCC model was successfully constructed, as reported by the previous studies [37, 60, 61]. The present study demonstrated that Fe_3_O_4_/Cs treatment causes significant decreases in ALT, AST, and GGT levels. Also, inflammation response and pathological changes were alleviated by Fe_3_O_4_/Cs. Such results come in agreement with the previous studies [42, 62].

The present study concluded that administration of Fe_3_O_4_/Cs effectively inhibited the phosphorylations of PI3K/Akt/mTOR pathway and modulated P38, ERK, and JNK pathways. Moreover, it causes apoptosis in cancer cells by increasing the expression of cleaved caspase-3 and induces immunosuppression for cytokines. In addition, the inflammation response and pathological changes were alleviated by Fe_3_O_4_/Cs.

## Data Availability

The data presented in this study are available in this article.
